# Outcomes of Autologous and Synthetic Bone Grafts in Locking Plate Fixation of Comminuted Distal Radius Fractures

**DOI:** 10.7759/cureus.60595

**Published:** 2024-05-19

**Authors:** Dhivakaran Gengatharan, Walter Wong, Dawn S Chia

**Affiliations:** 1 Orthopaedic Surgery, Sengkang General Hospital, Singapore, SGP; 2 Hand and Reconstructive Microsurgery, Tan Tock Seng Hospital, Singapore, SGP

**Keywords:** fracture comminution, fracture malunion, locking plate implants, bone graft, distal radius fractures

## Abstract

Background

The introduction of locking plate technology has improved the feasibility of distal radius fracture fixation without the need for bone grafting, yet challenges persist in cases of severely comminuted fractures and small, unstable intra-articular fragments. This study aimed to assess the outcomes of bone grafting in severely comminuted distal radius fractures treated with locking plates.

Methods

We performed a retrospective analysis involving 450 patients who underwent distal radius fracture fixations. We evaluated wrist motion, grip strength, and radiographic parameters, including radial inclination, radial tilt, ulnar variance, articular step, and fracture union at standardized intervals. In addition, at the 12- and 24-month marks, we assessed the disabilities of the arm, shoulder, and hand (DASH) questionnaire score.

Results

Out of the 450 patients who underwent distal radius fracture fixation using volar locking plate systems, 59 individuals (13%) required either autologous bone graft (n = 24) or synthetic bone substitutes (n = 35). In the final follow-up, all fractures had successfully united, displaying an average volar tilt of 4°, radial inclination of 18.8°, and an articular step or gap of 0.1 mm.

Conclusion

There was no significant difference between the use of autologous or synthetic bone grafts on clinical or radiological outcomes in the long term. Bone grafts are useful in severe metaphyseal comminution and aid in the reduction of articular fragments and bi-cortical comminution.

## Introduction

Distal radius fractures are a common condition encountered in emergency services, constituting up to 18% of all fractures [1]. These fractures exhibit a bimodal distribution, manifesting in young individuals subjected to high-energy trauma and more commonly in elderly patients with osteoporotic bones resulting from low-energy trauma. Approximately 40% of encountered distal radius fractures are deemed unstable, contributing to a considerable economic burden. Notably, the average cost of care for distal radius fractures in the United States was reported to reach as high as eight thousand dollars between 2009 and 2015 [2,3].

The advent of locking plates in the 2000s marked a significant milestone in fracture management, and over the years, this technology has proven to be an enduring and transformative force in principles of fracture fixation. Locking plates have revolutionized the approach to fracture fixation by introducing a mechanism that enhances resistance to implant loosening and screw pull-out. This innovation departs from traditional non-locking plate systems, as locking plates no longer rely on the compression of the plate to the bone. This design results in a more robust and stable fixation, reducing dependence on friction between the plate and the bone and providing superior stability, particularly in osteoporotic bone [4-7].

Locking plate technology has gained popularity in the fixation of distal radius fractures due to its versatility in addressing metaphyseal and articular fragments. Consequently, it is deemed suitable for challenging scenarios, such as fractures with short articular fragments, comminuted fractures requiring a bridging implant, periprosthetic fractures, and fixation of osteoporotic bone [8]. The management of comminuted distal radius fractures using earlier non-locking implants typically necessitated supplementary bone grafting. However, the advent of locking plate technology has significantly diminished the requirement for concurrent bone grafting during the definitive fixation of such comminuted fractures.

No official practice guidelines are available to recommend when bone grafts are indicated in distal radius fractures [9]. Current general indications for bone grafting include moderate to severe defects [10-12] and harnessing the osteoconductive, osteoinductive, and osteogenic properties of bone grafts to promote bony healing [10,13-17]. In view of decreasing the need for bone grafting in comminuted distal radius fracture fixation with the introduction of the locking plate fixation technique, we decided to evaluate the relevance of bone grafting in the locking plate fixation of distal radius. This article aims to study the results of bone grafting in distal radius fractures fixed with locking plates. Our secondary aim was to study the differences between the use of autologous grafts and bone graft substitutes in locking plate fixation of distal radius fractures.

## Materials and methods

We conducted a retrospective analysis of a group of consecutive, non-randomized patients obtained from the distal radius fracture registry at Tan Tock Seng Hospital, Singapore. The study spanned a period of 18 months, during which we identified a total of 1,106 distal radius fractures. Among these, 450 fractures underwent operative treatment, and 59 of those involved bone grafting with locking plate fixation.

Inclusion criteria encompassed acute distal radius fractures treated with locking implants and the insertion of bone grafts, either autografts or synthetic calcium phosphate-based substitutes. Autologous bone grafts were harvested from sites, such as the distal radius, distal ulna, olecranon, and the iliac crest during the same surgical procedure. Patients presenting with multiple fractures, or with concomitant limb ischemia at the time of admission, and malunions for corrective osteotomy were excluded from the study. Fifty-nine consecutive patients meeting the inclusion criteria were identified to form the study group.

Surgery was performed under either general or regional anesthesia. Fixation of the fracture was performed by the senior authors. Locking plate and screw distal radius fixation systems were used to hold the final fixation alignment. The indications for locking plate fixation system were indicated for patients with osteoporotic bone, presence of fracture comminution, and location of fracture being juxta and intraarticular. The reasons for bone graft utilization were divided into categories of metaphyseal comminution, structural support for bicortical defects, and intraarticular depression from die punch fracture fragments. An intraoperative image intensifier aided the fracture reduction and fixation of the fracture alignment and reduction. Bulky soft dressings were applied without the use of backslab stabilization after surgery.

Following surgery, all patients underwent standardized assessments at regular intervals and participated in a rehabilitation program guided by hand occupational therapists. Postoperative data were prospectively collected at weeks 2, 6, and 12 and subsequently at months 3, 6, 12, and 24. Post-surgical evaluations included clinical, radiological, and functional assessments. Measurements encompassed range of motion, grip strength, radiographic union, and parameters such as radial height, inclination, dorsal tilt, and ulnar variance. Functional scoring utilizing the disabilities of the arm, shoulder, and hand (DASH) and the Green O’Brien questionnaire scores were employed. The rehabilitation protocol involved postoperative protected interval mobilization within a wrist splint.

The collected data were analyzed using STATA 15 software (StataCorp. 2017, College Station, TX: StataCorp LLC) to assess the findings.

## Results

Bone grafts were utilized in 59 out of 450 (13%) distal radius fixations, with patients averaging 55 years old (ranging from 16 to 96 years old). The study comprised 27 male and 32 female patients, and the average follow-up period was 15 months, ranging from six to 31 months. Fractures were classified using the AO classification system, and the distribution of fractures requiring bone grafting is detailed in Table 1.

**Table 1:** Distribution of fractures requiring bone grafting based on the AO classification for distal radius fractures.

		Total	Autologous bone graft	Synthetic bone graft
Number of patients		59	24	35
Gender				
	Male	27	9	18
	Female	32	15	17
Age		55	56 (17-81)	54 (16-96)
Fracture classification				
	A1	0		
	A2	3		
	A3	11		
	B1	1		
	B2	1		
	B3	3		
	C1	4		
	C2	10		
	C3	26		
Indications for bone grafts				
	Dorsal metaphyseal comminution	13		
	Bicortical defect/ structural support	29		
	Intra-articular depression	17		

Locking plate fixation was performed in 31 fractures using a volar approach, 9 with a dorsal approach, and 19 with a combined volar and dorsal approach.

Indication for bone grafting

Indications for bone grafts included significant metaphyseal (volar/dorsal/combined) comminution affecting the maintenance of reduction, bi-cortical defects, and severely comminuted and depressed articular fragments. Autologous sources provided bone grafts in 24 cases, while commercially available bone graft substitutes were used in 35 cases. The olecranon was the most common source for autologous bone grafts (Table 2).

**Table 2:** Description of the fracture patterns that were indications for bone grafts used and the source of bone grafts obtained.

Source of bone grafts		Number
Autologous		24
	Olecranon	19
	Iliac crest	3
	Distal radius	1
	Ulna	1
Synthetic		35

**Figure 1:**
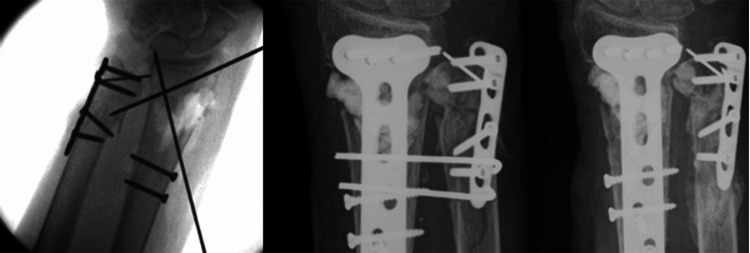
Metaphyseal comminution with a large segment of bone loss. K wires did not adequately hold the temporary reduction of the fracture. A synthetic bone graft was used to fill the bone void and maintain the stability of reduction for fixation. (Lt: intra-op fluoro showing metaphyseal defect; Middle: immediate post-op; Rt: four months)

**Figure 2:**
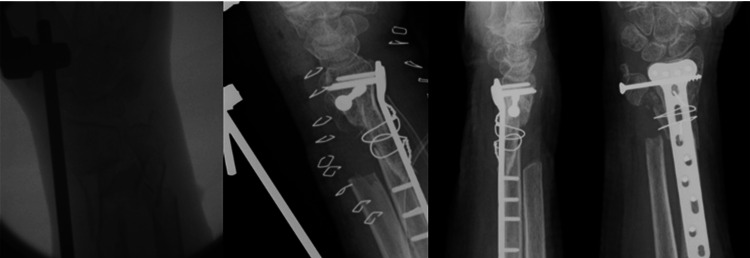
Large metaphyseal defect bridged with a corticocancellous autologous iliac crest graft shows union at three months. Lt to Rt: intra-op fluoro showing metaphyseal defect, immediate post-op, AP + Lat at three months

**Figure 3:**
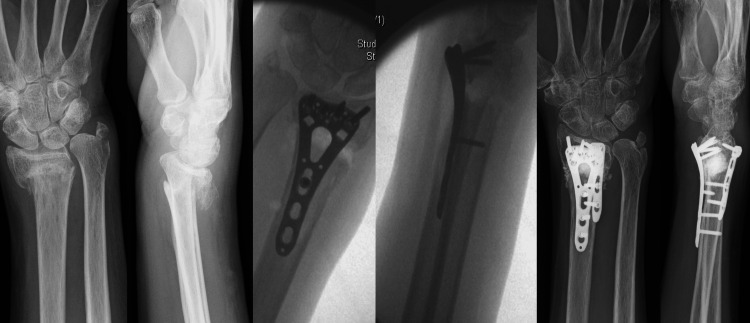
Metaphyseal bicortical defect with fracture shortening. Bone graft in the form of a bone block was used to restore the radial height and stabilization of the fracture reduction before the application of the locking plate.

Examples of applications of bone grafts are illustrated in Figures 1-3. 

Radiological outcomes

**Figure 4:**
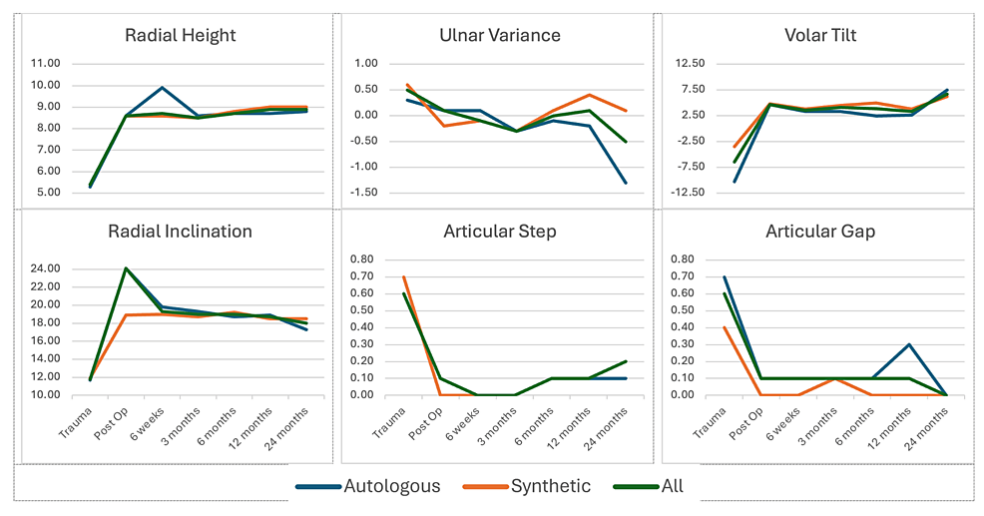
Series of graphs showing the radiological outcomes (radial height, ulnar variance, volar tilt, radial inclination, articular step, and articular gap) obtained over time.

Radiological outcomes, including union, reduction, and graft resorption, were favorable. All fractures achieved union, with an average time to union of two months (range four to 16 weeks). No cases resulted in non-union requiring re-grafting, and radiological parameters were consistently maintained over time (Figure 4).

Intra-articular fractures did not exhibit loss of articular reduction, leading to significant intra-articular step and gap. There were no notable differences in time to union or maintenance of fracture reduction when comparing outcomes of autologous and synthetic bone grafts. Radiological arthritis was detected in nine out of the 59 patients in which we utilized bone grafts. Among these cases, five patients had received autologous bone grafts, while four patients had received synthetic bone grafts.

Clinical and functional outcomes

**Figure 5:**
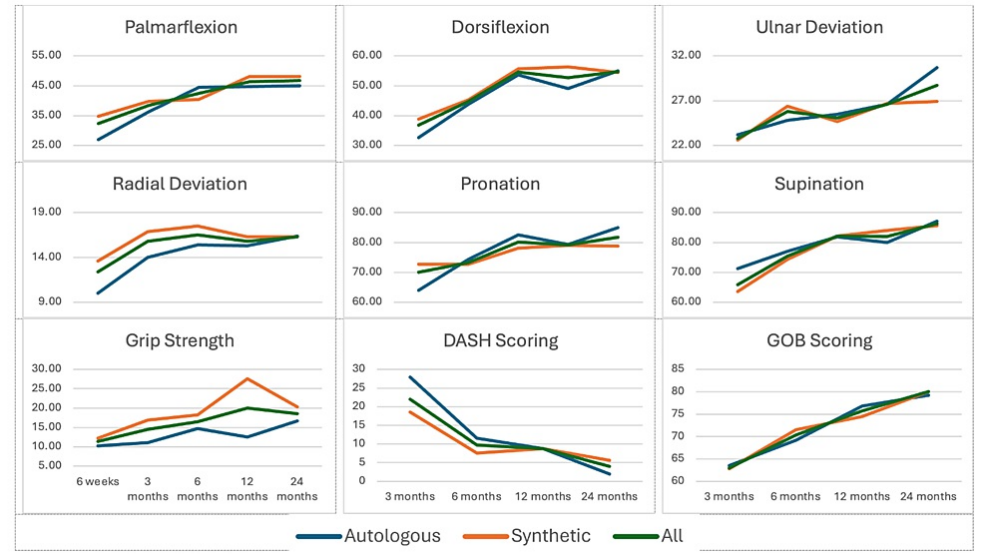
The series of graphs showing the components of clinical outcomes achieved in each direction of movement (palmarflexion, dorsiflexion, ulnar deviation, radial deviation, pronation, and supination) and grip strength, and the patient reported functional scores using the DASH and Green O’Brien scores measured over time. DASH: disabilities of the arm, shoulder, and hand

Clinical and functional outcomes demonstrated that the achieved average range of motion was sufficient for daily living activities (Figure 5). There was no statistical difference in the results between the autologous and synthetic bone graft groups. The DASH and the Green O'Brien scores showed improvement over time. At the 12- and 24-month marks, the average DASH score was 9 and 4, respectively, and 78% of the patients exhibited good to excellent function at 24 months. There was no significant donor site morbidity observed in patients who underwent autologous bone grafting. 

## Discussion

The introduction of the locking plate fixation system has revolutionized the scope of fractures amenable to surgical fixation. The distal periarticular screws, functioning both as an angular stable platform and fragment-specific screws, have contributed to improved fixation of complex fracture configurations. The latest generation of locking plate implants, featuring polyaxial variable angle designs, has further enhanced the management of intricate fractures, such as those involving dorsal comminution or defects. Notably, the use of locking plates has led to a decreased reliance on bone grafts in the fixation of distal radius fractures. Opening wedge osteotomy of malunited fractures and fractures with dorsal comminution or defects fixed with such locking plates were observed to unite, despite the presence of the defect. It hence appeared that bone grafting was not essential in the fixation of distal radius fractures, and bone graft utilization has decreased with the use of locking plates [18-21].

The American Academy of Orthopedic Surgery (AAOS) Treatment of Distal Radius Guidelines and Cochrane Systematic Review highlights inconclusive evidence regarding the use of supplemental bone grafts during locking plate fixation of distal radius fractures [9,22]. Mori et al. concluded in their study on the use of bone substitutes in the elderly who underwent volar locking plate fixation that there was also no significant difference in the clinical and radiological outcomes, but it was associated with higher medical costs, and they recommended the indications be considered [23]. Chang et al. did not find that bone graft augmentation was necessary if intraoperative anatomical reduction was achieved [24].

Despite the advantages of locking technology systems, bone grafting has not been rendered obsolete. Our study indicated an incidence of 13% of distal radius fractures fixed with locking plates required intraoperative bone grafting. This is similar to a prospective multicentre trial by Jupiter et al. studying the use of 2.4 mm locking plates, which found a similar overall incidence of 12% of fixations requiring bone graft supplementation during fracture fixation [25], demonstrating that surgeons continue to require bone grafts as an essential part of the distal radius fixation toolkit. When we analyzed the situations in which bone grafts were used by the surgeon, specific intraoperative scenarios were highlighted and categorized into situations where there was segmental metaphyseal structural loss, severely comminuted metaphyseal fractures, and elevation of depressed articular fragments. Bone grafts can still be beneficial to serve as cortico-cancellous tricortical bone blocks to bridge and maintain the reduction of comminuted fractures before implant fixation, particularly in the context of comminuted fractures as they lack reduction memory. For the severely comminuted metaphysis, the bone graft takes on the role of a scaffold to bridge the gap segment entirely when a tricortical iliac crest bone graft is employed. In a similar manner, bicortical (combined volar and dorsal) defects require bone grafts as struts as reduction in length is near impossible without the use of a bone graft or an external fixation as a distractor.

Locking plates may not be sufficient to maintain the length and reduction in the presence of large metaphyseal defects. Therefore, we advocate the use of autologous structural grafts for addressing large metaphyseal defects and comminution. In the case of depressed intra-articular fragments, after the articular fragments are reduced against the lunate, a bone graft was inserted through the fracture site packed into the metaphyseal bone, and a cartilage undersurface acts as a subchondral platform to support and maintain the position of the articular fragment and prevent relapse of the preoperative fracture gap and step. Bone grafts act as a structural support aiding in maintaining the position of reduced fragments after reduction. By augmenting the reduction, the stability of comminuted fractures was improved.

In our observations, there were no discernible differences between the utilization of synthetic and autologous bone grafts in terms of the time to incorporation and union of the fracture. Radiological, clinical, and functional outcomes were also comparable between both groups in both short term and long term. This is in agreement with Tosti’s conclusion that bone grafting may improve overall radiographic alignment and short-term outcomes although it has no significant long-term advantage [26].

In addition, our study directly compared the use of autologous and synthetic grafts, which has not been previously investigated as comparative study groups. Goto and colleagues investigated the use of artificial bone grafts (intraporous calcium hydroxyapatite IP-CHA) in volar locking plate fixation of osteoporotic distal radius fractures and found that although there were no significant differences in clinical outcomes, hydroxyapatite bone graft substitute might be effective for supporting the reduction of comminuted fractures and preventing secondary displacement after surgery [27]. Pace et al. found the use of autologous bone graft preferable to synthetic grafts for corrective osteotomies for distal radius fracture malunions. However, the sample size of synthetic grafts was too small to conclusively support this [28]. Jakubietz et al. studied the utility of granular bone graft substitutes in dorsal plating of comminuted fractures and showed no statistically significant difference in the clinical or radiological outcomes between the groups postoperatively when bone graft was either used or omitted and no difference in the occurrence of secondary fixation failure between the two groups [29]. In a prospective randomized study, a comparison was made between cancellous allograft and iliac crest bone autograft for the treatment of comminuted distal radius fractures. The results indicated no discernible differences in terms of pain or function when both groups were assessed one year post-surgery. However, it was noteworthy that the autograft group exhibited a significantly higher rate of complications [30]. 

Hence, we recommend that the selection of autologous or synthetic bone graft is better guided by the purpose of the bone graft to suit the specific fracture fixation need. It is crucial to note that synthetic bone grafts lack the osteogenic and osteoinductive properties inherent in autologous bone grafts. As a result, they may be better suited for applications, such as rafting or supporting articular fragments, rather than for use in bridging cortical defects. The selection of graft constitution as a corticocancellous, block, or granular form should be tailored to the indicative utility of the graft in order to fulfill fracture complexity.

The study acknowledges a limitation related to its retrospective study nature. Further investigations based on the patient's age, bone quality, and the type of trauma should be considered as these may have an impact on bone graft behavior.

## Conclusions

Bone grafting continues to be a crucial component of distal radius fracture fixation, even with the advent of locking plate fixation. It is employed in cases characterized by severe metaphyseal comminution and depressed articular fragments. Our findings indicate that there are no substantial disparities in outcomes when comparing the use of autologous and synthetic bone grafts. The primary role of bone grafts lies in facilitating the reduction of articular fragments and addressing bi-cortical comminution, contributing predominantly to short-term reduction. However, over the long term, their impact on clinical or radiological outcomes is not found to be statistically significant.

## References

[1] Nellans KW, Kowalski E, Chung KC (2012). The epidemiology of distal radius fractures. Hand Clin.

[2] Chung KC, Watt AJ, Kotsis SV, Margaliot Z, Haase SC, Kim HM (2006). Treatment of unstable distal radial fractures with the volar locking plating system. J Bone Joint Surg Am.

[3] Huetteman HE, Zhong L, Chung KC (2018). Cost of surgical treatment for distal radius fractures and the implications of episode-based bundled payments. J Hand Surg Am.

[4] Kubiak EN, Fulkerson E, Strauss E, Egol KA (2006). The evolution of locked plates. J Bone Joint Surg Am.

[5] Miller DL, Goswami T (2007). A review of locking compression plate biomechanics and their advantages as internal fixators in fracture healing. Clin Biomech (Bristol, Avon).

[6] Gautier E, Perren SM, Cordey J (2000). Effect of plate position relative to bending direction on the rigidity of a plate osteosynthesis. A theoretical analysis. Injury.

[7] Stoffel K, Dieter U, Stachowiak G, Gächter A, Kuster MS (2003). Biomechanical testing of the LCP--how can stability in locked internal fixators be controlled?. Injury.

[8] Hunt S, Buckley R (2013). Locking plates: a current concepts review of technique and indications for use. Acta Chir Orthop Traumatol Cech.

[9] Lichtman DM, Bindra RR, Boyer MI (2011). American Academy of Orthopaedic Surgeons clinical practice guideline on: the treatment of distal radius fractures. J Bone Joint Surg Am.

[10] Leung KS, Shen WY, Leung PC, Kinninmonth AW, Chang JC, Chan GP (1989). Ligamentotaxis and bone grafting for comminuted fractures of the distal radius. J Bone Joint Surg Br.

[11] Sanders R, Keppel F, Waldrop J (1991). External fixation of distal radial fractures: results and complications. J Hand Surg Am.

[12] Rikli DA, Küpfer K, Bodoky A (1998). Long-term results of the external fixation of distal radius fractures. J Trauma.

[13] Thielemann FW, Spaeth G, Veihelmann D, Schmidt K (1982). Osteoinduction. Part I: test model and comparative long term observation of allogenic and xenogenic matrix implants. Arch Orthop Trauma Surg (1978).

[14] Simmons DJ, Sherman NE, Lesker PA (1974). Allograft induced osteoinduction in rats. A circadian rhythm. Clin Orthop Relat Res.

[15] Koskinen E, Ryppy S, Lindholm T (1972). Osteoinduction and osteogenesis in implants of allogeneic bone matrix: influence of somatotropin, thyrotropin, and cortisone. Clin Orthop Relat Res.

[16] Lewandrowski KU, Tomford WW, Schomacker KT, Deutsch TF, Mankin HJ (1997). Improved osteoinduction of cortical bone allografts: a study of the effects of laser perforation and partial demineralization. J Orthop Res.

[17] Ozer K, Chung KC (2012). The use of bone grafts and substitutes in the treatment of distal radius fractures. Hand Clin.

[18] Knirk J, Jupiter J (1986). Intra-articular fractures of the distal end of the radius in young adults. J Bone Joint Surg Am.

[19] Palmer AK, Werner FW, Murphy D, Glisson R (1985). Functional wrist motion: a biomechanical study. J Hand Surg Am.

[20] Ryu JY, Cooney WP, Askew LJ, An KN, Chao EY (1991). Functional ranges of motion of the wrist joint. J Hand Surg Am.

[21] Gesensway D, Putnam MD, Mente PL, Lewis JL (1995). Design and biomechanics of a plate for the distal radius. J Hand Surg Am.

[22] Handoll HH, Watts AC (2008). Bone grafts and bone substitutes for treating distal radial fractures in adults. Cochrane Database Syst Rev.

[23] Mori Y, Takegami Y, Tokutake K, Oka Y, Imagama S (2023). Retrospective comparative study of clinical outcomes and cost-effectiveness with bone substitutes on volar locking plate fixation of unstable distal radial fractures in the elderly. J Hand Surg Asian Pac Vol.

[24] Chang FS, Chen CH, Lee CH, Lee KT, Cho YC (2020). Evaluating the necessity of bone augmentation for distal radius fracture fixed with a volar locking plate: a retrospective study. BMC Musculoskelet Disord.

[25] Jupiter JB, Marent-Huber M (2009). Operative management of distal radial fractures with 2.4-millimeter locking plates. A multicenter prospective case series. J Bone Joint Surg Am.

[26] Tosti R, Ilyas AM (2010). The role of bone grafting in distal radius fractures. J Hand Surg Am.

[27] Goto A, Murase T, Oka K, Yoshikawa H (2011). Use of the volar fixed angle plate for comminuted distal radius fractures and augmentation with a hydroxyapatite bone graft substitute. Hand Surg.

[28] Valerio Pace, Pasquale Sessa, Matteo Guzzini (2021). Clinical, functional and radiological outcomes of the use of fixed angle volar locking plates in corrective distal radius osteotomy for fracture malunion. Acta Biomed.

[29] Jakubietz MG, Gruenert JG, Jakubietz RG (2011). The use of beta-tricalcium phosphate bone graft substitute in dorsally plated, comminuted distal radius fractures. J Orthop Surg Res.

[30] Rajan GP, Fornaro J, Trentz O, Zellweger R (2006). Cancellous allograft versus autologous bone grafting for repair of comminuted distal radius fractures: a prospective, randomized trial. J Trauma.

